# YouTube™ as a Source of Information on Acupuncture for Correction of Breech Presentation

**DOI:** 10.7759/cureus.35182

**Published:** 2023-02-19

**Authors:** Alessandro Libretti, Daniela Surico, Christian Corsini, Carmen Imma Aquino, Sara Fracon, Valentino Remorgida

**Affiliations:** 1 Obstetrics and Gynaecology, University Hospital Maggiore della Carità, Novara, ITA; 2 Division of Experimental Oncology/Unit of Urology, Urological Research Institute, Istituto di Ricovero e Cura a Carattere Scientifico, San Raffaele University Hospital, Milan, ITA; 3 Anesthesia and Critical Care, University Hospital Maggiore della Carità, Novara, ITA

**Keywords:** pregnancy, obstetrics, breech, youtube, breech presentation, acupuncture

## Abstract

Background and Aim: Breech presentation is a condition that occurs in rare cases in pregnancy. Although guidelines recommend a cesarian section or an external cephalic version in case of breech, alternative procedures like acupuncture, are also available. Information on this approach is mostly found by patients through social media; we aimed to study content quality and the reliability of information present on YouTube™ (Google LLC, Mountain View, California, United States), one of the most popular.

Methods: Two gynecologists and an anesthesiologist, who was qualified as an acupuncturist, rated the reliability and the content quality of 23 of the first 100 results from YouTube. Normal data distribution was tested with the Shapiro-Wilk test. General features of videos, reliability, and content quality were compared with the Wilcoxon-Mann-Whitney test (continuous variables) and the Chi-square test (categorical variables). All tests were two-sided, and the statistical significance level was determined at p<0.05.

Results: Concerning reliability, all videos were rated poorly while only one was judged as sufficiently high in quality content. Lower scores in terms of reliability and content quality resulted from the reviewers' evaluation with no videos reported as suggestable to patients. Two videos were considered fit to be suggested to patients by the gynecologist reviewers.

Conclusions: Information about the role and the success rate of acupuncture for converting breech presentation found on YouTube are poorly reliable, low-quality, and not valid for patients. It should be a physician's duty to provide correct information to patients.

## Introduction

Breech presentation is a condition in which the fetus lies longitudinally with feet or buttocks closest to the cervix. There are three different types of breech: frank, complete, and kneeling or footling [1]. The incidence of breech presentation decreases with the advancing of the gestation and it’s lower after the 37th week of pregnancy; it occurs in almost 3-5% of pregnancies [1]. There are various factors associated with breech presentation and the main are: uterine congenital malformations, myomas, oligohydramnios, preterm delivery, small-for-gestational-age fetus, and some fetal malformations [2].

Perinatal morbidity and mortality of infants in breech presentation at the end of pregnancy are higher than in cephalic presentation for injuries such as clavicle fractures, hematomas, and brachial plexus traumas while the cerebral palsy rate is not different [1,2]. Programming a cesarean section at term can reduce but not abolish perinatal mortality, attested to be around 1% [3]. When vaginal delivery is not contraindicated for other reasons (e.g., placenta previa), techniques to convert breech presentation and avoid breech labor are proposed by healthcare professionals. Among these are: external cephalic version, acupuncture, moxibustion (a Chinese medicine therapy that consists of burning mugwort on various points on the body), and postural methods like knee-chest position and supine hip elevation [4]. The external cephalic version, with a success rate of around 50% [4], is a maneuver where the gynecologist, under ultrasound control, tries to rotate the fetus into a head-down position by applying pressure through the mother’s abdominal wall [5]. The overall complication rate of this maneuver ranges from 1-2% [6]. Even though rare, complications like umbilical cord entanglement, abruptio placentae, preterm labor, and premature rupture of membranes, besides maternal discomfort, have been reported [6]. According to guidelines, the external cephalic version is the only procedure that can be recommended to patients, preferably after the 37th week of gestation [4,6]. Controversial data can be found in the literature about the effectiveness of alternative methods such as moxibustion and acupuncture in turning breech presentation [7-10]. 

Acupuncture is a traditional Chinese medicine procedure that involves the activation by a fine needle of the acupoint ‘Bladder 67 or Zhiyin point’, which is located beside the toenail of the fifth toe [9-11]. The aim of this study was to record the perception of the information available to patients on the internet. We selected YouTube™ (Google LLC, Mountain View, California, United States) as this well-known video-sharing website is commonly used by patients as a source of information about healthcare [12], and surveys have confirmed that it has the potential to be an important vehicle for disseminating medical information [13-15].

In this study, we evaluated, using two scales, the reliability and the quality of the videos suggested by the YouTube algorithm to a hypothetical patient who is looking for information about the acupuncture treatment proposed for the condition of breech baby.

## Materials and methods

Video selection

We queried https://www.youtube.com/ in November 2022 using the following keywords: “breech presentation acupuncture”. The YouTube setting was “global”, and no filters were used to mimic the search strategy of a hypothetical patient proposed with acupuncture to turn breech presentation. We limited our search to the first 100 videos as studies indicate that only 8% of users of the internet continue their search after the third page of results [15]. The inclusion criteria of videos were that they had to contain both information about acupuncture and its use in breech presentation. The study didn’t require ethics committee approval as no human subjects or participants were involved and the videos were publicly accessible. After watching the first 100 videos (for a total of 12 hours), we selected 23 videos that met the inclusion criteria.

The full playlist of the 23 selected videos is freely available on youtube @alessandrolibretti6557. Please note that one video is not present in the playlist because YouTube does not allow videos from the "Short" category to be added to playlists.

Data extraction

We extracted data from each of the 23 selected videos. Data extracted were: (i) Language (all videos but one were in English, one was in Italian), (ii) Production source: videos from experts (healthcare professionals or acupuncture institutes), videos from internet or tv, and videos from patients (without professional support), (iii) Aims of videos (informative versus sponsor; videos were classified as "sponsor" when a commercial purpose emerged, together or not with references to a center or healthcare professionals, while videos were classified as informative when aimed to provide only information on acupuncture and breech without a clear sponsor purpose), (iv) Duration of upload on YouTube (months), (v) Video length (seconds), (vi) Number of views, (vii) Number of likes, (viii) Number of comments, and (ix) Number of subscribers of the uploader.

Assessment of reliability and content quality

The ranking of videos has been calculated using two scales of quality evaluation by three experts: two experienced gynecologists and one experienced anesthesiologist who was qualified as acupuncturist. To guarantee the scientific reliability of the videos, we used the modified DISCERN scale, a scale used for the assessment of written health information (mDISCERN) [16]. To assess the score of the mDISCERN, experts had to answer the following five questions and eventually assign a point to each one: (i) Are the aims clear and achieved? (ii) Are reliable sources of information used? (iii) Are the information presented balanced and unbiased? (iv) Are additional sources of information listed for patient references? (v) Are areas of uncertainty mentioned?

The reliability of the health information was reached when the mDISCERN total score was 3 or more. To evaluate the content quality of the videos, the Global Quality Scale (GQS) was used. This scale was originally developed to evaluate the fluency and the easiness of online information [17]. To assert the score of the GQS, experts had to choose one of the following sentences to classify the video: (i) Low quality, low flow, most information missing, not beneficial for patients, (ii) Usually low quality and low flow of information, some listed information and many important issues are missing, very limited use for patients, (iii) Moderate quality, insufficient flow of information, some important information are sufficiently discussed but some are poorly discussed; only somewhat useful for patients, (iv) Good quality and generally good information flow, most of the relevant information is listed, but some topics are not covered, useful for patients, and (v) Excellent quality and information flow, very useful for patients.

A higher GQS score indicates better content of the videos. Three reviewers (VR, DS, and SF) assessed the reliability of information and the content quality of the videos. They watched these videos and assigned marks for each question asked, independently from the others but at the same time, during a four-day session. The experts were also asked a final question: Would you suggest this video to a patient of yours?

Statistical analysis

The normal distribution of data was tested with the Shapiro-Wilk test. Data were presented as medians (interquartile range (IQR)) for continuous variables and frequencies (proportions) for categorical variables. Data from general features and results of the assessment of the videos were presented as mean, standard deviation (SD), and minimum (min)-maximum (max) for each variable. Data were presented stratified according to the production source (experts vs. others), mean mDISCERN, mean GQS, and aim of the video (informative vs. sponsor). General features of the videos, reliability of the content of the video, and global quality were compared among the groups with the Wilcoxon-Mann-Whitney test for continuous variables and the Chi-square test for categorical variables, respectively. Statistical analyses were performed using Rstudio integrated development environment for R software v. 3.5.3 (2016; RStudio, Boston, Massachusetts, United States). All tests were two-sided, and the statistical significance level was determined at p < 0.05.

## Results

Of the 100 videos, 23% met the inclusion criteria and were selected for the review. General features of the selected videos (duration of upload, length, views, likes, number of subscribers to the uploader) and results of the assessment (mDISCERN and GQS scores according to the reviewers) are presented in Table 1.

**Table 1:** General features and results of the assessment of the videos

Video features	Mean ± SD (Min, Max)
Duration of upload on YouTube (months)	59.49 ± 37.99 (12.83, 145.20)
Video length (seconds)	592.20 ± 1033.70 (39.00, 4834.00)
Number of views	26824 ± 55420.36 (16, 249589)
Number of likes	147.86 ± 339.30 (0, 1277)
Number of subscribers of the uploader	217997 ± 745279.4 (6, 3580000)
mDISCERN score	1.48 ± 1.06 (0, 3.67)
Gynecologists	1.94 ± 1.35 (0, 4.50)
Anesthesiologist	0.56 ± 0.84 (0, 2)
Global Quality Scale score	2.03 ± 0.82 (1, 3.67)
Gynecologists	2.33 ± 1.06 (1, 4.5)
Anesthesiologist	1.44 ± 0.66 (1, 3)

Of the 23 included videos, 16 (69,5%) were produced by experts (10 by professionals, six by acupuncture institutes), seven (30,5%) were produced by non-experts, three by patients, three by television, and one by a social media influencer. The mean mDISCERN score for the selected videos was 1.48±1.06. It was 1.94±1.35 when assigned by the two gynecologists and 0.56±0.84 when assigned by the anesthesiologist/acupuncturist. The mean difference in the mDISCERN score between the two categories of specialists was 1.38. Moreover, the highest score assigned by the gynecologists was 4.5 while the anesthesiologist/acupuncturist assigned a maximum of 2.

No videos were unanimously rated as reliable (mDISCERN) by the three experts although four videos were rated as reliable by the two gynecologists. The mean GQS score for the selected videos was 2.03±0.82. It was 2.33±1.06 when assigned by the two gynecologists and 1.44±0.66 when assigned by the anesthesiologist/acupuncturist. The mean difference in the GQS score between the two categories of specialists was 0.89. Nevertheless, the highest score assigned by gynecologists was 4.5 while the anesthesiologist/acupuncturist assigned a maximum of 3. While one of the gynecologists (VR) judged two videos to be of excellent quality (classified as 5 on the GQS scale), none of the 23 videos obtained the same classification by the other two reviewers. Only one video was judged as sufficiently high in quality content (GQS) unanimously by the three experts. While the anesthesiologist/acupuncturists did not suggest any videos to patients, two videos were simultaneously approved to be suggested to patients by the gynecologists.

The comparison of general features and results of the assessment of the videos among groups according to production source (experts versus non-experts) is presented in Table 2.

**Table 2:** Comparison of the general features and results of the assessment of the videos among the groups according to the production source IQR: inter quartile range; NaN: not applicable

	Expert (Professionals=10, Acupuncture institutes=6)	Other (Television=3, Patients=3, Social Media=1)	p-value
Numbers	16	7	
Duration of upload on YouTube (month), median (IQR)	46.47 (26.07, 67.61)	75.13 (40.34, 122.20)	0.18
Video length (seconds), median (IQR)	119.00 (81.25, 781.00)	252.00 (157.50, 552.00)	0.64
Number of views, median (IQR)	2,166.50 (179.00, 19,244.75)	4,364.00 (893.50, 36,924.50)	0.66
Number of comments, median (IQR)	0.00 (0.00, 4.00)	1.00 (0.00, 3.00)	0.78
Number of likes, median (IQR)	10.50 (1.00, 29.50)	21.00 (9.25, 414.50)	0.21
Number of subscribers, median (IQR)	662.00 (148.25, 7,507.50)	61,100.00 (11,410.00, 267,000.00)	0.08
mDISCERN, median (IQR)	1.33 (0.92, 2.08)	1.67 (0.67, 2.17)	0.87
mDISCERN scores			1.00
<3, n (%)	15 (93.8)	6 (85.7)	
>/=3, n (%)	1 (6.2)	1 (14.3)	
Global Quality Scale total, median (IQR)	2.10 (0.86)	1.86 (0.77)	0.52
Global Quality Scale scores			0.98
<3, n (%)	12 (75.0)	6 (85.7)	
>/=3, n (%)	4 (25.0)	1 (14.3)	
Suggest to patients			1.00
Gynecologists YES, n (%)	1 (6.2)	1 (14.3)	
Gynecologists NO, n (%)	15 (93,8)	6 (85.7)	
Anesthesiologist YES, n (%)	0 (0)	0 (0)	NaN

Although a classification according to production source (experts versus non-experts) was made, no statistically significant differences (p<0.05) were found among the general features of the videos. The three experts assigned similar scores independent of who produced the videos. Videos produced by experts weren't more likely to be suggested to their patients by the reviewers than those produced by non-experts. Among the 23 videos published on YouTube, 13 were categorized as "informative" while 10 were categorized as "sponsor". The comparison of general features of videos according to their purpose is presented in Table 3.

**Table 3:** Comparison of general features among the groups according to the aim of the videos. IQR: inter quartile range; NaN: not applicable

	Informative	Sponsored	p-value
Numbers	13	10	
Duration of upload on YouTube (months), median (IQR)	39.80 (27.47, 53.10)	63.13 (56.03, 81.03)	0.22
Video length (seconds), median (IQR)	769.00 (147.00, 879.00)	94.00 (44.00, 274.75)	0.01
Number of views, median (IQR)	929.00 (116.00, 15,963.00)	7,832.50 (1,997.75, 27,144.25)	0.58
Number of comments, median (IQR)	0.00 (0.00, 3.25)	1.00 (0.00, 4.50)	0.62
Number of likes, median (IQR)	10.00 (4.00, 34.00)	12.00 (3.00, 28.00)	0.84
Numbers of subscribers, median (IQR)	5,810.00 (494.00, 255,000.00)	662.00 (263.00, 9,615.50)	0.21
mDISCERN score, median (IQR)	1.67 (1.00, 2.67)	1.00 (0.00, 1.58)	0.03
mDISCERN scores			0.581
<3, n (%)	11 (84.6)	10 (100.0)	
>/=3, n (%)	2 (15.4)	0 (0.0)	
Global Quality Scale total, median (IQR)	2.31 (0.82)	1.67 (0.70)	0.06
Global Quality Scale scores			0.49
<3, n (%)	9 (69.2)	9 (90.0)	
>/=3, n (%)	4 (30.8)	1 (10.0)	
Suggest to patients			0.58
Gynecologists YES, n (%)	2 (15.4)	0 (0)	
Gynecologists NO, n (%)	11 (84.6)	10 (100)	
Anesthesiologist YES, n (%)	0 (%)	0 (%)	NaN
Production source			0.28
Hospital, n (%)	4 (30.8)	2 (20.0)	
Social Media, n (%)	0 (0.0)	1 (10.0)	
Patients, n (%)	3 (23.1)	0 (0.0)	
Professionals, n (%)	4 (30.8)	6 (60.0)	
Television, n (%)	2 (15.4)	1 (10.0)	

Informative videos were longer than sponsor videos (p=0.01). The median mDISCERN score obtained by informative videos was higher, 0.67 points, than the score obtained by sponsor videos (p=0.03). No additional differences were found in other features according to the videos' purpose.

A comparison of the general features among groups according to the mDISCERN score is presented in Table 4. The mDISCERN score was similar according to the general features of videos when assigned by the gynecologists and the anesthesiology/acupuncturist. No additional differences were found in other features according to the mDISCERN score.

**Table 4:** Comparison of the general features among groups according to the mDISCERN score IQR: inter quartile range; NaN: not applicable

	mDISCERN <3	mDISCERN >/=3	p-value
Numbers	21	2	
Duration of upload on YouTube (month), median (IQR)	50.03 (32.65, 81.03)	71.81 (43.47, 100.16)	1.00
Video length (seconds), median (IQR)	147.00 (84.00, 769.00)	2,503 (1,334.50, 3,667.50)	0.23
Number of views, median (IQR)	2,978.00 (116.00, 31,094.00)	8,254 (4,398.75, 12,108.25)	0.91
Number of comments, median (IQR)	1.00 (0.00, 4.00)	0 (0, 0)	0.18
Number of likes, median (IQR)	11.50 (2.50, 38.50)	13.01 (10.00, 16.00)	1.00
Number of subscribers, median (IQR)	662.00 (154.00, 24,500.00)	142,405 (74,107.50, 210,702.50)	0.23
Suggest to paitents			0.39
Gynecologists YES, n (%)	1 (4.8)	1 (50.0)	
Gynecologists NO, n (%)	20 (95.2)	1 (50.0)	
Anesthesiologist YES, n (%)	0 (0)	0 (0)	NaN
Production			0.514
Hospital, n (%)	6 (28.6)	0 (0.0)	
Social Media, n (%)	1 (4.8)	0 (0.0)	
Patients, n (%)	3 (14.3)	0 (0.0)	
Professionals, n (%)	9 (42.9)	1 (50.0)	
Television, n (%)	2 (9.5)	1 (50.0)	

A comparison of general features among groups according to the GSQ score is presented in Table 5. As in the mDISCERN score, the GQS score was the same according to the general features of videos when assigned by the gynecologists and the anesthesiology/acupuncturist. No additional differences were found in other features according to the GQS score.

**Table 5:** Comparison of the general features among the groups according to the GQS score. IQR: inter quartile range; NaN: not applicable; GQS: Global Quality Scale

	GSQ <3	GSQ >/=3	p-value
Numbers	18	5	
Duration of upload on YouTube (month), median (IQR)	53.10 (32.93, 81.03)	39.80 (31.80, 106.60)	0.97
Video length (seconds), median (IQR)	134.00 (80.75, 493.50)	817 (168.00, 1,390.00)	0.12
Number of views, median (IQR)	2,324.50 (116.00, 25,962.00)	15,295 (1,355.00, 15,963.00)	0.33
Number of comments, median (IQR)	0.00 (0.00, 2.50)	4.00 (0.00, 4.00)	0.38
Number of likes, median (IQR)	10.00 (1.00, 26.00)	34.01 (19.00, 52.00)	0.08
Number of Subscribers, median (IQR)	662.00 (136.75, 23,575.00)	12,600 (5,810.00, 217,000.00)	0.10
Suggest			0.06
Gynecologists YES, n (%)	0 (0)	2 (40.0)	
Gynecologists NO, n (%)	18 (100)	3 (60.0)	
Anesthesiologist YES, n (%)	0 (0)	0 (0)	NaN
Production			0.77
Hospital, n (%)	5 (27.8)	1 (20.0)	
Social Media, n (%)	1 (5.6)	0 (0.0)	
Patients, n (%)	3 (16.7)	0 (0.0)	
Professionals, n (%)	7 (38.9)	3 (60.0)	
Television, n (%)	2 (11.1)	1 (20.0)	

## Discussion

**Figure 1:**
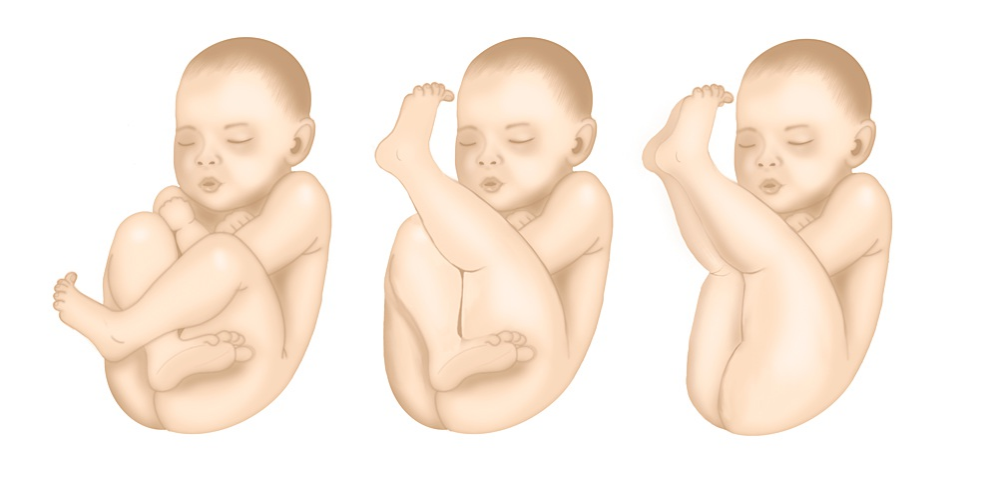
Types of breech presentation: complete breech, incomplete breech (kneeling or footling), frank breech Figure commissioned by A. Libretti, exclusively for this article

There is a huge number of videos addressing the topic of acupuncture and its efficacy in turning breech babies (Figure 1) presented on YouTube. That given, only one video of the 23 analyzed was judged as sufficiently high in quality content unanimously, while no one was judged sufficiently valid to be suggested to patients nor rated as reliable by all three experts. The anesthesiologist/acupuncturist was stricter in rating videos, and we can speculate asserting that this could be secondary to her major expertise and direct experience with the acupuncture technique. Interestingly, no differences in evaluating the reliability were obtained according to the production source (professionals or acupuncture institutes). Similarly, videos produced by experts were not more frequently suggested by reviewers to their patients. Also, it was interesting that the quality of information available was not different between sponsor and informative videos. We observed that, on average, informative videos were longer and more reliable.

Our study is not devoid of limitations; although several studies are emerging to recognize the role and relevance of YouTube for health promotion and education of patients on specific conditions, a standardized way to assess these aspects is still missing. Despite this, to the best of our knowledge, the use of validated scales such as in this research, representing experts’ evaluation, is to date, according to the literature, the best way to define content quality and reliability. Moreover, as the volume of online videos grows constantly, in our study we mimicked the search strategy of a hypothetical patient trying to figure out what she could find as prompted by the YouTube algorithm.

## Conclusions

YouTube information on acupuncture and its use for breech presentation does not seem to be sufficiently valid for patients. Furthermore, the higher the expertise of the reviewers, the sterner the judgment of the information presented in the video.

As the access to YouTube and other mass media is constantly increasing, patients must be alerted that medical information present there could be inaccurate. While medical societies should be made responsible for providing correct public information, on the other hand, healthcare professionals should be aware that the information available through the internet are poor in quality and this could influence patients’ choices and perceptions.
